# Hepatic FOXA3 overexpression prevents Western diet–induced obesity and MASH through TGR5

**DOI:** 10.1016/j.jlr.2024.100527

**Published:** 2024-03-04

**Authors:** Raja Gopoju, Jiayou Wang, Xiaoli Pan, Shuwei Hu, Li Lin, Alyssa Clark, Yanyong Xu, Liya Yin, Xinwen Wang, Yanqiao Zhang

**Affiliations:** 1 Department of Integrative Medical Sciences, Northeast Ohio Medical University, Rootstown, OH, USA; 2 Department of Pharmaceutical Sciences, Northeast Ohio Medical University, Rootstown, OH, USA

**Keywords:** steatohepatitis, bile acids, obesity, lipids, liver

## Abstract

Forkhead transcription factor 3 (FOXA3) has been shown to regulate metabolism and development. Hepatic FOXA3 is reduced in obesity and fatty liver disease. However, the role of hepatic FOXA3 in regulating obesity or steatohepatitis remains to be investigated. In this work, C57BL/6 mice were i.v. injected with AAV8-ALB-FOXA3 or the control virus. The mice were then fed a chow or Western diet for 16 weeks. The role of hepatic FOXA3 in energy metabolism and steatohepatitis was investigated. Plasma bile acid composition and the role of Takeda G protein–coupled receptor 5 (TGR5) in mediating the metabolic effects of FOXA3 were determined. Overexpression of hepatic FOXA3 reduced hepatic steatosis in chow-fed mice and attenuated Western diet–induced obesity and steatohepatitis. FOXA3 induced lipolysis and inhibited hepatic genes involved in bile acid uptake, resulting in elevated plasma bile acids. The beneficial effects of hepatic FOXA3 overexpression on Western diet–induced obesity and steatohepatitis were abolished in *Tgr5*^−/−^ mice. Our data demonstrate that overexpression of hepatic FOXA3 prevents Western diet–induced obesity and steatohepatitis via activation of TGR5.

Hepatic triglyceride (TG) levels are regulated by de novo lipogenesis (DNL), fatty acid uptake, lipolysis, fatty acid oxidation (FAO), and VLDL secretion. Excessive TG deposition in the liver is the hallmark of metabolic dysfunction–associated steatotic liver disease (MASLD), which is an emerging healthcare issue and a major chronic liver disease worldwide. Disruption of hepatic fatty acid/TG homeostasis may cause lipotoxicity and the development of metabolic dysfunction–associated steatohepatitis (MASH) (1, 2). MASH is an advanced form of MASLD and is characterized by steatosis, hepatocyte ballooning, lobular inflammation, and fibrosis. So far, no Food and Drug Administration-approved drugs are available for the treatment of MASH.

Bile acids (BAs) play an important role in regulating intestinal fat absorption and metabolic homeostasis mainly via activating farnesoid X receptor and Takeda G protein–coupled receptor 5 (TGR5) (3, 4). Primary BAs are produced from cholesterol by cholesterol 7α-hydroxylase (CYP7A1) in the classic pathway and converted to secondary BAs by gut microbiota. Elevated total BAs and altered BA composition are observed in the plasma of MASLD and MASH patients (5). BA-activated TGR5 promotes thermogenesis and improves glucose homeostasis (6, 7, 8). TGR5 activation also inhibits the development of MAFLD/MASH (9, 10, 11, 12).

Forkhead box A3 (FOXA3) is a member of the winged-helix transcription factors. FOXA3 is expressed in the liver, intestine, pancreas, etc., and regulates glucose homeostasis, lipoprotein metabolism, and liver and pancreas development (13, 14, 15, 16, 17). Overexpression of hepatic FOXA3 is shown to protect against atherosclerosis by promoting reverse cholesterol transport (14). However, the role of hepatocyte FOXA3 in regulating diet-induced MASH has not been reported.

In the current study, we investigated the role of overexpression of hepatic FOXA3 in obesity and MASH development and the underlying mechanisms. Our data showed that overexpression of hepatic FOXA3 raised plasma BA levels and attenuated Western diet–induced obesity and MASH in WT mice but not in *Tgr5*^−/−^ mice. In addition, overexpression of hepatic FOXA3 also induced hepatic lipolysis and reduced hepatic TG levels in chow-fed mice.

## Materials and methods

### Mice and diets

C57BL/6J mice were purchased from the Jackson Laboratory (Bar Harbor, ME). *Tgr5*^−/−^ mice have been described previously (18). The Western diet (21% fat/0.2% cholesterol) was purchased from Envigo (cat# TD.88137). Unless otherwise stated, 2-month-old male mice were used and fed a Western diet for 16 weeks. The mice were fasted for 5–6 h during the light cycle before euthanasia. All the animal experiments were approved by the Institutional Animal Care and Use Committee at Northeast Ohio Medical University.

### Adeno-associated viruses

Human FOXA3 was cloned into an adeno-associated virus (AAV) vector under the control of a mouse albumin promoter to generate AAV-ALB-FOXA3 (14, 19). AAV8-ALB-Null (control) or AAV8-ALB-FOXA3 was produced and titrated by Vector BioLabs. Each mouse was i.v. injected with 3 × 10^11^ genome copies of AAVs.

### Quantitative real-time PCR

Total RNA was extracted from liver or brown adipose tissues (BATs) using Trizol reagent (Thermo Fisher Scientific), and mRNA levels were quantified by quantitative real-time PCR using Powerup SYBR Green Master mix (Thermo Fisher Scientific) on a 7500 real-time PCR machine (Applied Biosystems). mRNA levels were normalized to *36b4*.

### Hepatic lipids, hydroxyproline, and plasma ALT and AST

Approximately 100 mg of liver tissue was homogenized in methanol, and lipids were extracted using chloroform/methanol (2:1 v/v) as previously described (20). Hepatic TGs and total cholesterol (TC) were determined using Infinity reagents from ThermoFisher Scientific. Free cholesterol (FC) and FFAs were measured using Fujifilm Wako Chemicals. Hepatic hydroxyproline levels were measured using a kit from Cell Biolabs (cat # STA675). Plasma alanine transaminase (ALT) and aspartate transaminase (AST) levels were determined using Infinity reagents.

### Triglyceride hydrolase activity and FAO

Hepatic total cell lysates were isolated for measuring triglyceride hydrolase (TGH) activity using ^3^H-triolein as substrate (21). Primary hepatocytes were isolated and then cultured in Dulbecco’s modified Eagle’s medium containing 10% FBS in 12-well plates. FAO was analyzed using ^3^H-palmitic acid as substrate as previously described (21).

### Intestinal fat absorption

To determine intestinal fat absorption, mice were fasted for 6 h and then i.v. injected with tyloxapol (500 μg/kg) prior to gavage with olive oil (15 μl/g body weight) as described (19, 22). Plasma TG levels were determined at indicated time points.

### Western blotting

Total liver lysates and nuclear or microsome extracts of liver samples were used for Western blotting. The antibodies against FOXA3/HNF3γ A-2 (cat # sc-74424) and histone (cat # sc-393358) were purchased from Santa Cruz Biotechnology. Antibodies against tubulin (cat # ab4074), NTCP (cat # ab131084), and OATP1A1 (cat # ab203036) were purchased from Abcam. Antibodies against CYP7A1 (cat # TA351400) and CYP8B1 (cat # TA313734) were purchased from Origene. Calnexin antibody (cat # NB1001965) was purchased from Novus Biologicals.

### BA levels, BA pool size, and BA composition

BA levels were determined using a BA kit from Diazyme (cat # DZ042A-KY1). BAs in the liver, gall bladder, and intestine were extracted using ethanol (23). Total BAs in the liver, gall bladder, and intestine were determined and used to calculate the total BA pool size. The BA composition was determined by LC-MS/MS.

### Glucose tolerance test

The glucose tolerance test was performed as previously described (24). Briefly, mice were fasted overnight, followed by i.p. injection of D-glucose (Sigma) at a dose of 1.8 mg/kg body weight. Plasma glucose levels at indicated time points were measured.

### Fatty acid composition and DNL

Hepatic fatty acid composition and DNL were analyzed using GC-MS as described previously (25, 26). For DNL, mice were given water containing 8% ^2^H_2_O for the last 7 days. Four hours prior to anesthesia, mice were i.p. injected with 30 μl/g ^2^H_2_O.

### Oil Red O, H&E, and picrosirius red staining

Liver tissues were fixed in 10% formalin and then embedded in paraffin or OCT. Liver sections were stained with Oil Red O, H&E, or picrosirius red. Images were acquired using an Olympus microscope.

### Body fat measurement, food intake, and energy expenditure

Body fat was measured using EchoMRI (EchoMRI, Houston, TX). Oxygen consumption, carbon dioxide production, and heat production were determined using Comprehensive Lab Monitoring System (Columbus Instruments) as described previously (27). In brief, mice underwent an acclimation period, and a 48-h measurement of energy expenditure was analyzed using an eight-chamber system. Each run included both the control and overexpression groups, with four mice per group. The energy expenditure data were analyzed using the web-based CalR program (28).

### Statistical analysis

All the data were expressed as mean ± SEM. Statistical significance was analyzed using an unpaired Student’s *t* test or ANOVA (GraphPad Prism, San Diego, CA). Differences were considered as statistically significant at *P* < 0.05.

## Results

### Hepatic overexpression of FOXA3 lowers hepatic TG accumulation in chow-fed mice

**Fig. 1:**
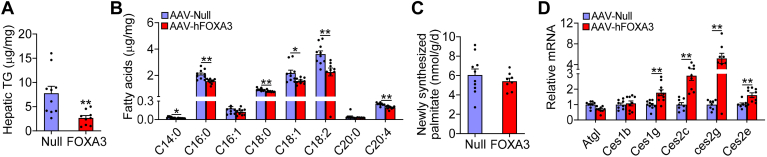
Hepatic overexpression of FOXA3 lowers hepatic triglyceride accumulation in chow-fed mice. C57BL/6 mice were i.v. injected with AAV8-ALB-Null or AAV8-ALB-FOXA3. After 4 weeks, hepatic triglyceride (TG) levels (A), fatty acid composition (B), newly synthesized palmitate levels (C), and mRNA levels (D) were determined (n = 10). A Student *t* test was used for statistical analysis. ∗*P* < 0.05 and ∗∗*P* < 0.01. AAV, adeno-associated virus; FOXA3, Forkhead box A3.

Previous studies show that hepatic FOXA3 is markedly reduced in genetic or high-fat diet–induced obesity and MASH patients (14). Since FOXA3 was expressed in mouse primary hepatocytes, AML12 cells, or HepG2 cells at a moderate level (supplemental Fig. S1A–C), we evaluated the role of hepatocyte FOXA3 in the regulation of hepatic lipid metabolism. C57BL/6 mice were i.v. injected with either AAV8-ALB-Null or AAV8-ALB-FOXA3, and then fed a chow diet for 4 weeks. Overexpression of FOXA3 had no impact on body weight (supplemental Fig. S2A, B) but caused a 66% reduction of hepatic TG levels (Fig. 1A), whereas hepatic FFA, TC, or FC levels remained unchanged (supplemental Fig. S2C, D). Interestingly, analysis of hepatic fatty acid composition by GC-MS showed that C14:0, C16:0, C18:0, C18:1, C18:2, and C20:4 fatty acid levels were significantly reduced in mice-overexpressing FOXA3 (Fig. 1B). Since hepatic fatty acid levels are determined by DNL, fatty acid uptake, FAO, fatty acid incorporation of TG, and TG hydrolysis (lipolysis), we analyzed hepatic DNL using GC-MS. There was no change in de novo synthesis of hepatic palmitate levels (Fig. 1C), suggesting that FOXA3 does not regulate DNL. Gene expression analysis showed that overexpression of hepatic FOXA3 significantly induced carboxylesterases *1g* (*Ces1g*), *Ces2c*, *Ces2g*, and *Ces2e* without affecting adipose TG lipase or *Ces1b* (Fig. 1D). We have previously shown that *Ces1g*/*Ces1* and *Ces2c*/*Ces2* have TG lipase (TGH) activity (21, 25), suggesting that FOXA3 may regulate lipolysis. In addition, hepatic overexpression of FOXA3 inhibited the expression of *Pparγ*, microsomal TG transfer protein, *Apob*, and inflammatory and fibrogenic genes (interleukin 1β and α-smooth muscle actin) (supplemental Fig. S2E). Together, these data indicate that hepatic FOXA3 prevents hepatic TG accumulation in chow-fed mice likely via inducing lipolysis.

### Hepatic overexpression of FOXA3 attenuates Western diet–induced obesity and steatohepatitis

**Fig. 2:**
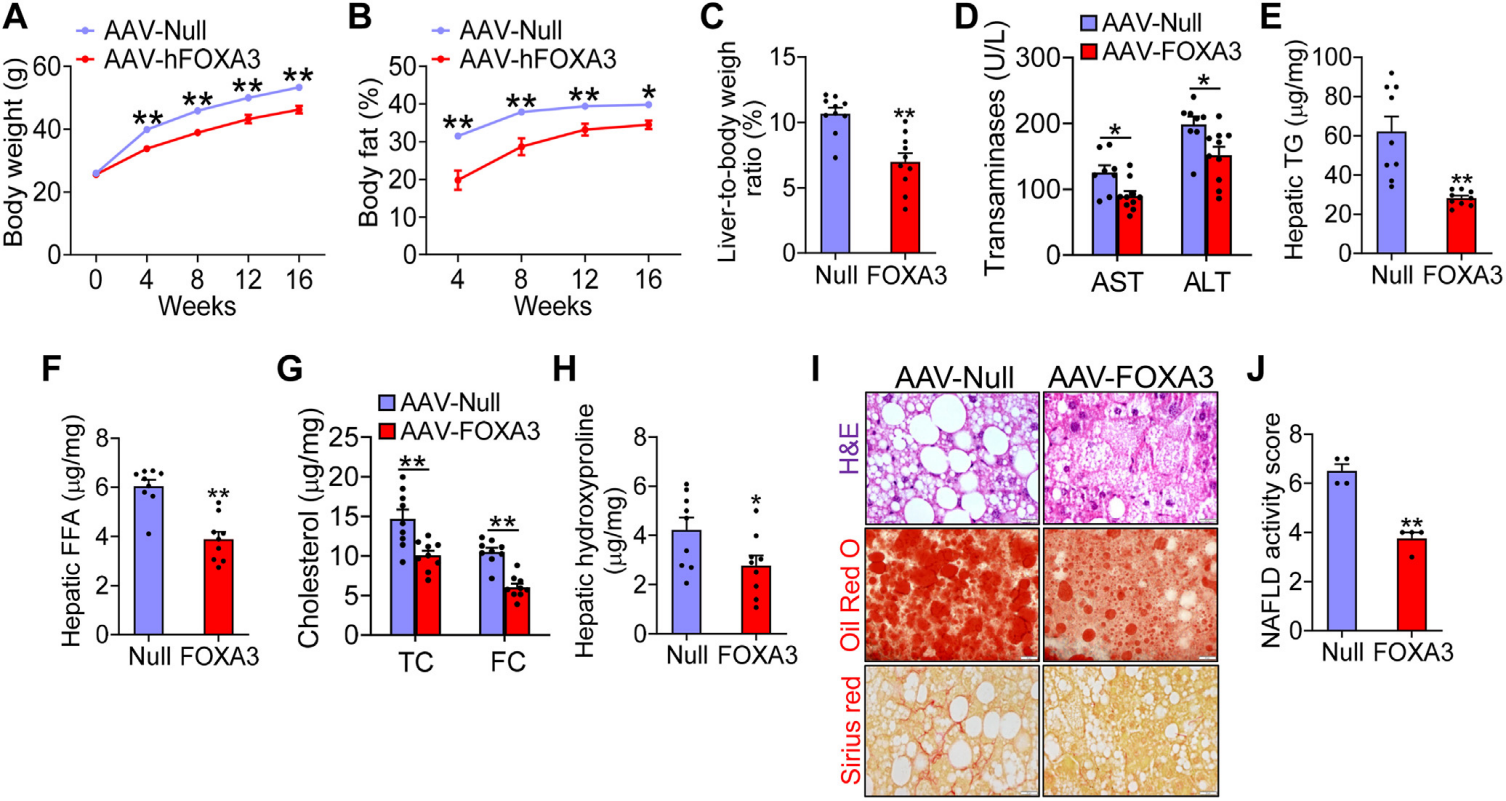
Hepatic overexpression of FOXA3 attenuates Western diet–induced obesity and steatohepatitis. C57BL/6 mice were i.v. injected with AAV8-ALB-Null or AAV8-ALB-FOXA3 and fed a Western diet for 16 weeks (n = 10). A: body weight. B: body fat content. C: liver-to-body weight ratio (%). D: plasma transaminase levels. E: hepatic triglyceride (TG) levels. F: hepatic FFA levels. G: hepatic total cholesterol (TC) and free cholesterol (FC) levels. H: hepatic hydroxyproline levels. I: histological staining of liver sections with H&E (top panel), Oil Red O (middle panel), or picrosirius red (bottom panel). J: NAFLD activity score. Scale bars in (I): 50 μm. A two-way ANOVA (A, B) or Student *t* test (C–H, J) was used for statistical analysis. ∗*P* < 0.05 and ∗∗*P* < 0.01. AAV, adeno-associated virus; FOXA3, Forkhead box A3.

When mice were fed a Western diet for 16 weeks, hepatic overexpression of FOXA3 led to a marked decrease in body weight (Fig. 2A), body fat content (Fig. 2B), and the ratio of the liver-to-body weight (Fig. 2C), without affecting glucose tolerance (supplemental Fig. S3A). Hepatic FOXA3 overexpression also significantly reduced plasma ALT and AST levels (Fig. 2D) and decreased hepatic TG levels by 55% (Fig. 2E), FFA levels by 36% (Fig. 2F), TC by 32%, FC by 43%, (Fig. 2G), and hydroxyproline by 35% (Fig. 2H). Consistent with the changes in biochemical parameters, histological staining by H&E, Oil Red O, or picrosirius red showed that hepatic FOXA3 overexpression reduced hepatocyte ballooning, hepatic neutral lipid accumulation, and fibrosis (Fig. 2I). As a result, hepatic FOXA3 overexpression reduced NAFLD activity score by 43% (Fig. 2J). Together, the data of Fig. 2 demonstrate that overexpression of hepatic FOXA3 attenuates Western diet–induced obesity and steatohepatitis.

### Overexpression of FOXA3 inhibits inflammation and induces lipolysis in the liver

**Fig. 3:**
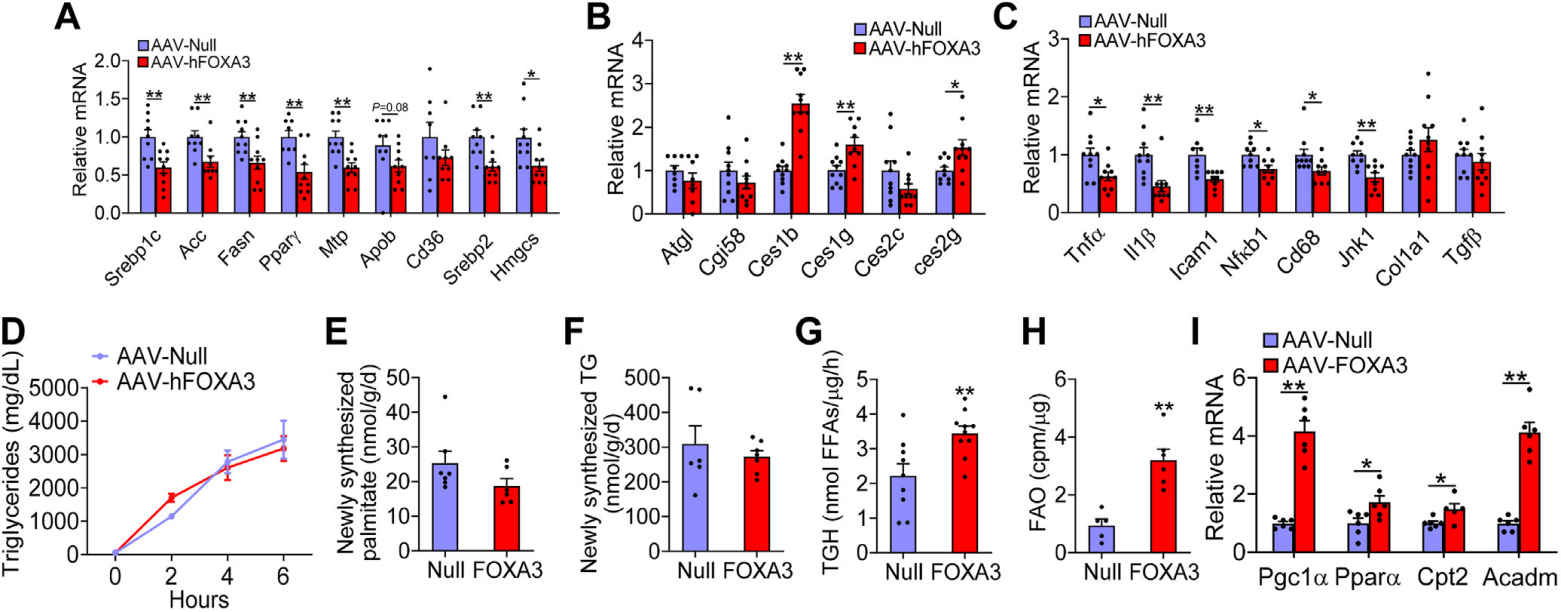
Hepatic overexpression of FOXA3 inhibits inflammation and induces lipolysis in the liver. C57BL/6 mice were i.v. injected with AAV8-ALB-Null or AAV8-ALB-FOXA3 and fed a Western diet for 16 weeks. A–C: hepatic gene expression (n = 9–10). D: intestinal fat absorption (n = 8–10). E: newly synthesized palmitate in the liver (n = 6–7). F: newly synthesized triglycerides (TGs) in the liver (n = 6–7). G: hepatic triglyceride hydrolase activity (TGH) (n = 9–10). H, I: primary hepatocytes were isolated. Fatty acid oxidation (FAO) (H) (n = 5) and mRNA levels (I) (n = 6) were determined. A two-way ANOVA (D) or a Student *t* test (A–C and E–I) was used for statistical analysis. ∗*P* < 0.05 and ∗∗*P* < 0.01. AAV, adeno-associated virus; FOXA3, Forkhead box A3.

To investigate how FOXA3 inhibited Western diet–induced steatohepatitis, we analyzed hepatic gene expression. Overexpression of hepatic FOXA3 significantly inhibited hepatic expression of lipogenic genes (*Srebp1c*, *Acc*, *Fasn*, *Pparγ*), VLDL secretion (microsomal TG transfer protein), and cholesterol synthesis (*Srebp2*, *Hmgcs2*), with a trend to decrease *Apob* expression and no change in *Cd36* expression (Fig. 3A). In contrast, hepatic FOXA3 overexpression significantly induced hepatic mRNA levels of *Ces1b*, *Ces1g/Ces1*, and *Ces2g*, whereas adipose TG lipase or *Cgi58* was unchanged (Fig. 3B). In addition, hepatic FOXA3 overexpression significantly inhibited hepatic proinflammatory genes, including *Tnfα*, interleukin 1β, *Icam1*, *Nfκb1*, *Cd68*, and *Jnk1*, without affecting *Tgfβ* or *Col1a1* expression (Fig. 3C).

Hepatic FOXA3 overexpression did not affect intestinal fat absorption (Fig. 3D). Interestingly, although FOXA3 inhibited lipogenic genes (Fig. 3A), hepatic newly synthesized palmitate levels (Fig. 3E) or newly synthesized TG levels (Fig. 3F) remained unchanged. Consistent with the induction of genes involved in lipolysis (Figs. 1D, 3B), overexpression of FOXA3-induced hepatic TGH activity by 55% (Fig. 3G) and increased FAO by 340% in primary hepatocytes (Fig. 3H). In line with the induction of FAO, overexpression of FOXA3 significantly induced the expression of *Ppar gamma* coactivator 1-alpha, *Pparα*, carnitine palmitoyltransferase II, and medium-chain acyl-CoA dehydrogenase (Fig. 3I). Thus, the data of Fig. 3 suggest that FOXA3 overexpression reduces diet-induced MASH development likely via inducing lipolysis and FAO and inhibiting inflammation.

### Overexpression of hepatic FOXA3 increases energy expenditure

**Fig. 4:**
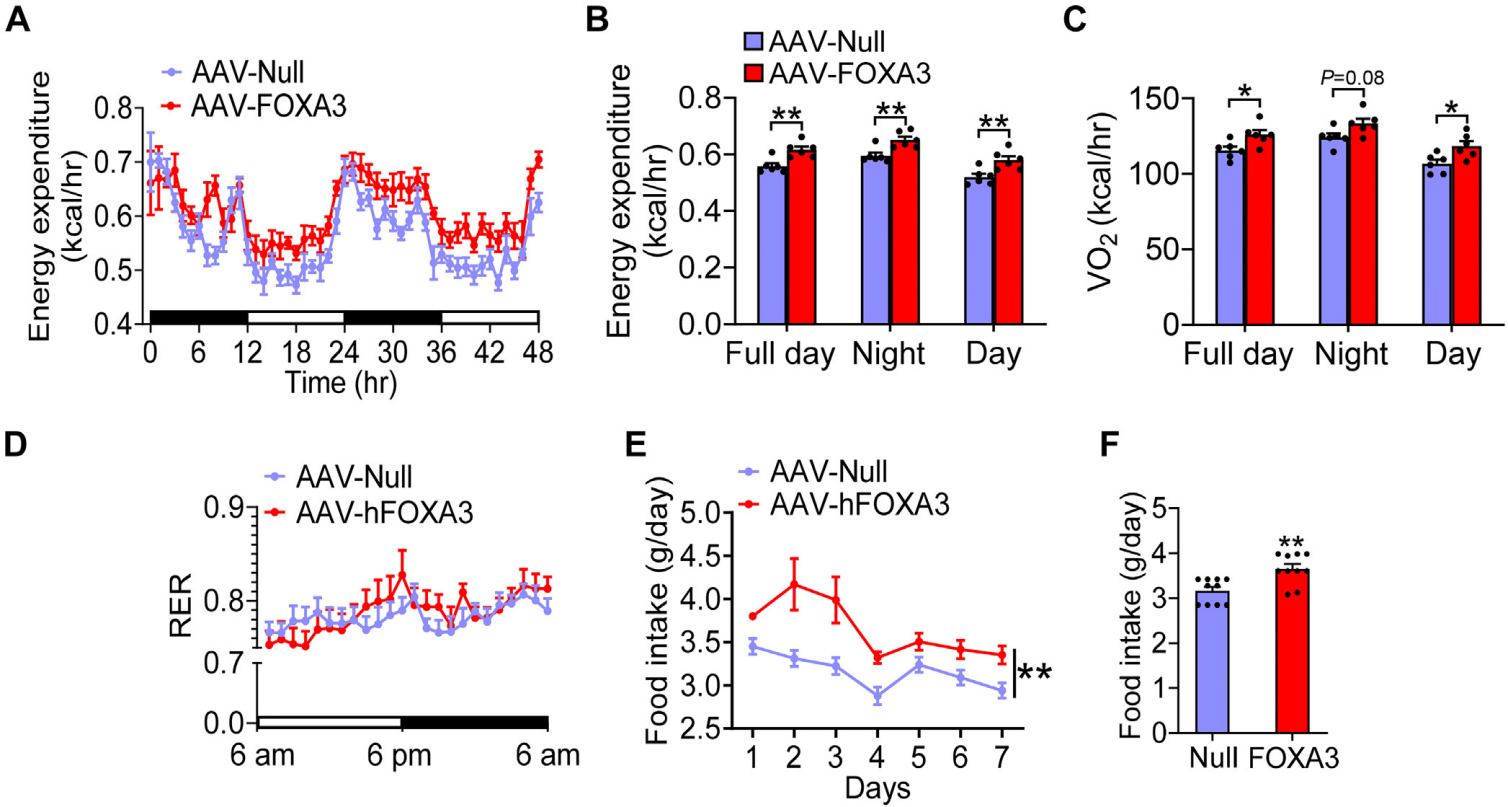
Overexpression of hepatic FOXA3 increases energy expenditure. C57BL/6 mice were i.v. injected with AAV8-ALB-Null or AAV8-ALB-FOXA3 and fed a Western diet for 16 weeks. A, B: energy expenditure over a 48-h period. C: VO_2_ during the night or daytime. D: respiration ratio (RER). E, F: food intake over 7 days. A two-way ANOVA (A–E) or a Student *t* test (F) was used for statistical analysis. ∗*P* < 0.05 and ∗∗*P* < 0.01. AAV, adeno-associated virus; FOXA3, Forkhead box A3.

To understand how overexpression of hepatic FOXA3 prevented Western diet–induced obesity, we analyzed energy expenditure using Comprehensive Lab Animal Monitoring System. Overexpression of hepatic FOXA3 significantly increased energy expenditure during the day and night time (Fig. 4A, B). Consistent with the increased energy expenditure, hepatic FOXA3–induced oxygen consumption (Fig. 4C) without affecting respiratory exchange ratio (Fig. 4D). Surprisingly, mice-overexpressing hepatic FOXA3 had increased food intake (Fig. 4E, F). Thus, hepatic FOXA3 overexpression attenuates Western diet–induced obesity via increasing energy expenditure.

### Overexpression of hepatic FOXA3 regulates BA metabolism

**Fig. 5:**
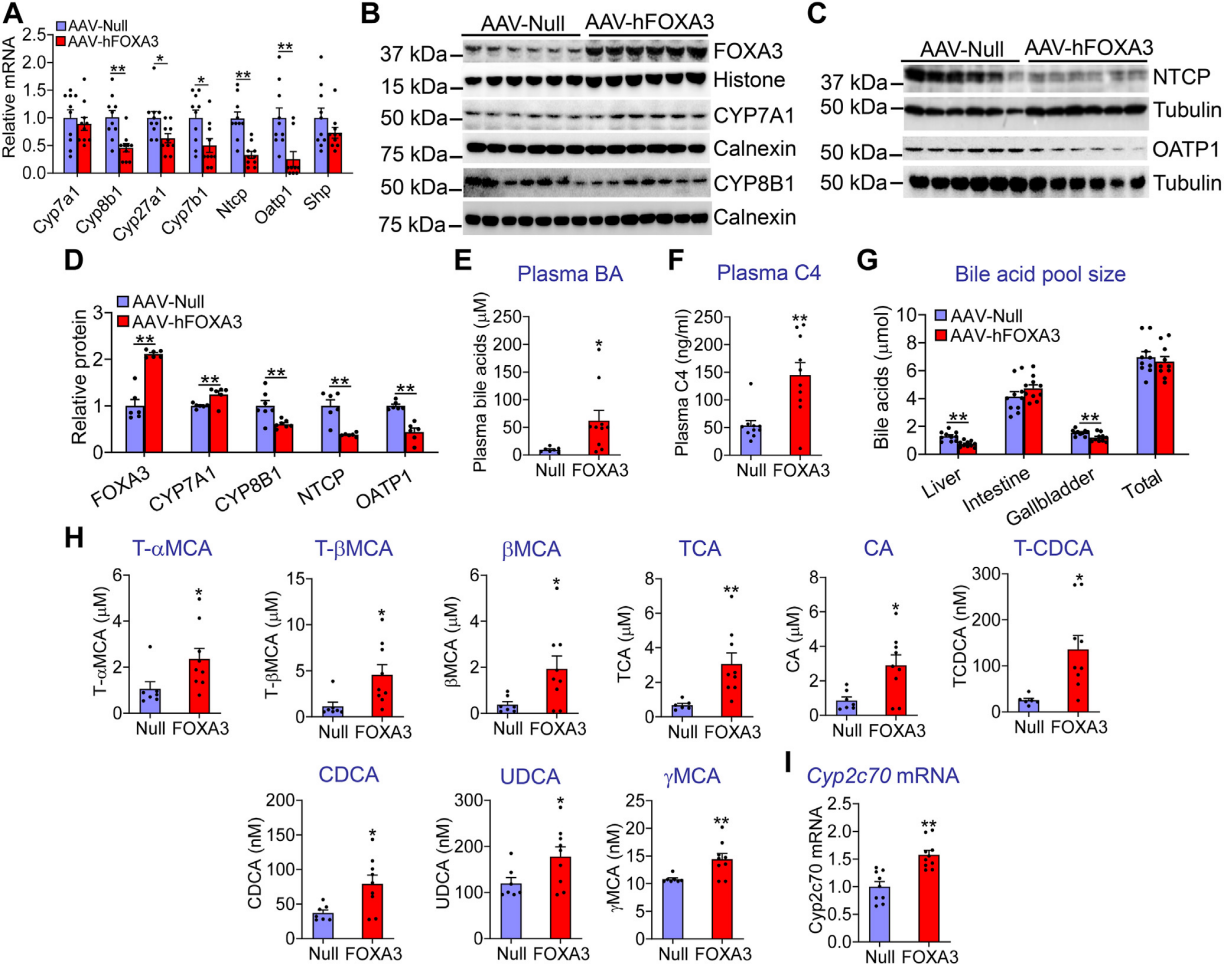
Overexpression of hepatic FOXA3 regulates bile acid (BA) metabolism. C57BL/6 mice were i.v. injected with AAV8-ALB-Null or AAV8-ALB-FOXA3 and fed a Western diet for 16 weeks (n = 6–9). A: hepatic mRNA levels. B, C: Western blot assays of hepatic proteins. D: relative hepatic protein levels. E: plasma bile acid levels. F: plasma C4 levels. G: BA pool size. H: individual bile acid levels in the plasma. I: hepatic *Cyp2c70* mRNA levels. A Student *t* test was used for statistical analysis. ∗*P* < 0.05 and ∗∗*P* < 0.01. AAV, adeno-associated virus; CYP7A1, cholesterol 7α-hydroxylase; FOXA3, Forkhead box A3.

BA signaling is known to regulate energy and lipid metabolism, but the role of FOXA3 in BA metabolism has not been reported before. Therefore, we investigated whether hepatic FOXA3 regulated BA metabolism. Hepatic FOXA3 overexpression reduced the mRNA levels of *Cyp8b1* by 54%, sterol 27-hydroxylase (*Cyp27a1*) by 37%, oxysterol and steroid 7α-hydroxylase (*Cyp7b1*) by 50%, Na^+^-taurocholate cotransporting polypeptide (*Ntcp*) by 67%, and organic anion transporting polypeptide 1 (*Oatp1*) by 75%, while hepatic *Cyp7a1* or small heterodimer partner mRNA levels were unchanged (Fig. 5A). NTCP and OATP1 play a key role in hepatic BA uptake from circulation (29). Western blot assays showed that hepatic overexpression of FOXA3-induced hepatic FOXA3 and CYP7A1 protein levels by 2.1-fold and 1.25-fold, respectively, and reduced hepatic CYP8B1, NTCP, and OATP1 protein levels by 40%, 72%, and 56%, respectively (Fig. 5B–D). There was no or a little change in hepatic expression of BA export transporter (*Bsep*), organic solute transporter alpha, organic solute transporter beta, BA-CoA:amino acid N-acyltransferase, BA-CoA synthase, multidrug resistance 2, multidrug resistance-associated protein 2, ABC transporters *Abcg5* or *Abcg8* (supplemental Fig. S3B). Consistent with the changes in genes involved in BA metabolism, overexpression of hepatic FOXA3 increased plasma total BA levels by 6.5-fold (Fig. 5E) and plasma 7-α-hydroxy-4-cholesten-3-one (C4) levels by 2.7-fold (Fig. 5F), and reduced BA levels in the liver and gallbladder, whereas BA levels in the intestine or total BA levels remained unchanged (Fig. 5G). Analysis of plasma BA composition by LC-MS/MS showed that overexpression of hepatic FOXA3 increased plasma levels of taura-conjugated α-muricholic acid by 2.3-fold, T-βMCA by 4-fold, βMCA by 5-fold, taura-conjugated cholic acid by 4.5-fold, CA by 3.3-fold, taura-conjugated chenodeoxycholic acid (T-CDCA) by 5.3-fold, CDCA by 2.1-fold, ursodeoxycholic acid by 1.5-fold, and γMCA by 1.3-fold (Fig. 5H). In line with the increased MCA levels, hepatic *Cyp2c70* mRNA levels were increased by 1.6-fold (Fig. 5I). Thus, overexpression of hepatic FOXA3 raises plasma BA levels likely through the inhibition of hepatic NTCP and OATP1 expression.

### TGR5 mediates FOXA3’s metabolic effects

**Fig. 6:**
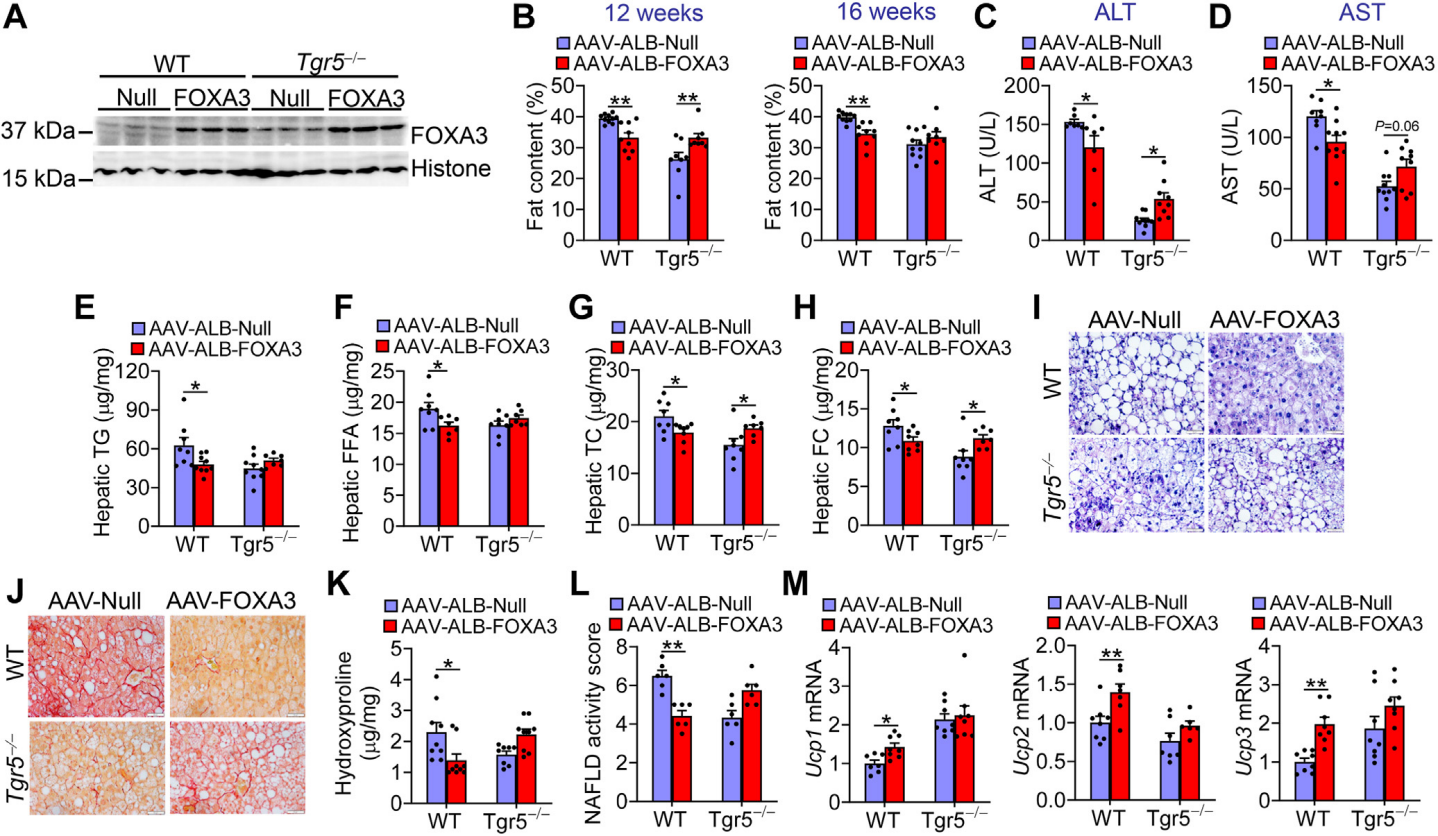
TGR5 mediates FOXA3’s metabolic effects. WT and *Tgr5*^−/−^ mice were i.v. injected with AAV8-ALB-Null or AAV8-ALB-FOXA3 and then fed a Western diet for 16 weeks (n = 8–10). A: Western blot assays of hepatic protein levels. B: body fat content at 12 weeks and 16 weeks. C: plasma ALT levels. D: plasma AST levels. E: hepatic triglyceride (TG) levels. F: hepatic FFA levels. G: hepatic total cholesterol (TC) levels. H: hepatic free cholesterol (FC) levels. I, J: H&E (I) or picrosirius red staining (J) of liver sections. K: hepatic hydroxyproline levels. L: NAFLD activity score. M: mRNA levels in brown adipose tissue. Scale bars in (I, J): 50 μm. A two-way ANOVA was used for statistical analysis. ∗*P* < 0.05 and ∗∗*P* < 0.01. AAV, adeno-associated virus; ALT, alanine transaminase; AST, aspartate transaminase; FOXA3, Forkhead box A3; TGR5, TGR5, Takeda G protein–coupled receptor.

The data of Fig. 5 show that plasma BA levels were significantly induced in mice-overexpressing hepatic FOXA3. BAs may activate TGR5 to induce thermogenesis (6, 8) and improve diet-induced MAFLD/MASH (9, 10, 11, 12). To investigate whether hepatic FOXA3 improves metabolic homeostasis via TGR5, we i.v. injected WT mice and *Tgr5*^−/−^ mice with AAV8-ALB-Null or AAV8-ALB-FOXA3 and then fed them a Western diet for 16 weeks. Overexpression of hepatic FOXA3 (Fig. 6A) decreased body weight gain in WT mice but not in *Tgr5*^−/−^ mice (supplemental Fig. S4A, B) and reduced body fat content at 12 weeks and 16 weeks in WT mice but not in *Tgr5*^−/−^ mice (Fig. 6B). Similarly, overexpression of hepatic FOXA3 reduced plasma levels of ALT (Fig. 6C) and AST (Fig. 6D) as well as hepatic levels of TG (Fig. 6E), FFAs (Fig. 6F), TC (Fig. 6G), and FC (Fig. 6H) in WT mice, but not in *Tgr5*^−/−^ mice. H&E and picrosirius red staining showed FOXA3 overexpression reduced hepatic lipid accumulation and fibrosis in WT mice, but not in *Tgr5*^−/−^ mice (Fig. 6I, J). Consistent with these data, overexpression of hepatic FOXA3 reduced hepatic hydroxyproline (Fig. 6K) and NAFLD activity score (Fig. 6L) in WT mice, but not in *Tgr5*^−/−^ mice.

Gene expression data show that overexpression of hepatic FOXA3 induced uncoupled protein 1 (*Ucp1*), *Ucp2*, and *Ucp3* expression in BAT of WT mice, but not in *Tgr5*^−/−^ mice (Fig. 6M). Hepatic FOXA3 overexpression had no impact on *Dio2* or *Cpt1a* expression in BAT (supplemental Fig. S4C, D), but induced *Cpt1b* or *Cd36* expression in BAT in an *Tgr5*-dependent manner (supplemental Fig. S4E, F). Taken together, these studies indicate that activation of the TGR5 signaling is required for hepatic FOXA3 to ameliorate Western diet–induced obesity and hepatic steatosis.

## Discussion

Previous studies have shown that hepatic FOXA3 expression is markedly reduced in obesity or MASH (14). However, whether hepatic FOXA3 regulates diet-induced obesity or MASH has not been investigated. In this project, we show that overexpression of hepatic FOXA3 reduces hepatic TG accumulation in chow-fed mice and ameliorates Western diet–induced obesity and MASLD/MASH. Mechanistically, hepatic FOXA3 induces lipolysis and thermogenesis and prevents Western diet–induced metabolic disorders via TGR5.

One important finding of this study is that overexpression of hepatic FOXA3 raises plasma BA levels by inhibition of hepatic NTCP and OATP1 expression. NTCP is expressed exclusively in the liver (30). Previous studies by Donkers *et al.* (31) show that loss of NTCP prevents diet-induced obesity and hepatic steatosis by inducing energy expenditure and inhibiting intestinal fat absorption. Our data show that hepatic FOXA3 improves diet-induced obesity and MASH in WT mice, but not in *Tgr5*^−/−^ mice, suggesting that activation of TGR5 plays a key role in mediating the beneficial effects of FOXA3. BAs activate TGR5 in the order of potency of LCA>DCA>CDCA>CA with an EC50 of 0.53 μM, 1.0 μM, 4.4 μM, and 7.7 μM, respectively (32, 33). Activated TGR5 induces thermogenesis and improves diet-induced MAFLD/MASH (6, 8, 9, 10, 11, 12). Interestingly, Donkers *et al.* (31) show that loss of NTCP or both NTCP and TGR5 has similar effects on obesity or hepatic steatosis, suggesting that loss of NTCP exerts beneficial effects independent of activation of TGR5. It is unclear whether OATP1 is compensatively induced in *Ntcp*-deficient mice. In our studies, FOXA3 inhibits both NTCP and OATP1, which may allow a drastic increase in plasma BA levels and thus activation of TGR5. It remains unclear how the loss of NTCP leads to increased energy expenditure (31).

In MASH patients, plasma BA levels are generally increased by ∼2- to 3-fold (5, 34, 35). Interestingly, the increase in plasma BAs does not or is not sufficient to protect against MASH development. Grzych *et al.* (35) show that plasma BA levels are elevated only in MASH patients with pronounced insulin resistance, suggesting that the increase in plasma BAs in MASH may result from liver damage. In contrast, FOXA3 overexpression raises plasma BA levels by 6.5-fold via the inhibition of NTCP and OATP1, which appears to be sufficient to activate TGR5 to exert beneficial metabolic effects.

In addition to raising plasma BA levels by inhibition of NTCP and OATP1, FOXA3 overexpression also induces hepatic TGH activity likely through increasing the expression of CESs, such as CES1 and CES2, which have been demonstrated to have TGH activity (21, 25). The increased lipolytic activity may account for reduced hepatic TG levels in chow-fed mice-overexpressing hepatic FOXA3, as body weight is not changed in these mice. FOXA3 also induces FAO and inhibits SREBP2, which, together with reduced obesity, may account for reduced hepatic FFA and FC levels in Western diet–fed mice. A reduction in hepatic FFA and FC levels may attenuate hepatic lipotoxicity, resulting in reduced hepatic inflammation and fibrosis (1, 2).

In summary, we have identified a novel role of hepatic FOXA3 in regulating lipid and BA metabolism, obesity, and MASH. Since hepatic FOXA3 is markedly reduced in obesity and MASH, targeting hepatic FOXA3 may be a useful strategy for the treatment of obesity and MASH.

## Data availability

All the data are contained within the manuscript.

## Supplemental data

This article contains supplemental data.

## Conflict of interest

The authors declare that they have no conflicts of interest with the contents of this article.
