# Supplementary material for: Hepatic FOXA3 overexpression prevents Western diet–induced obesity and MASH through TGR5
Source: J Lipid Res. 2024 Mar 4;65(4):100527. doi: 10.1016/j.jlr.2024.100527 (PMC10999823; doi:10.1016/j.jlr.2024.100527)
Supplement: Supplemental Figures S1–S4 [file mmc1.pdf]

Supplementary Information

Hepatic FOXA3 overexpression prevents Western diet-induced obesity and MASH through TGR5

Raja Gopoju, Jiayou Wang, Xiaoli Pan, Shuwei Hu, Li Lin, Alyssa Clark, Yanyong Xu, Liya Yin, Xinwen Wang, Yanqiao Zhang

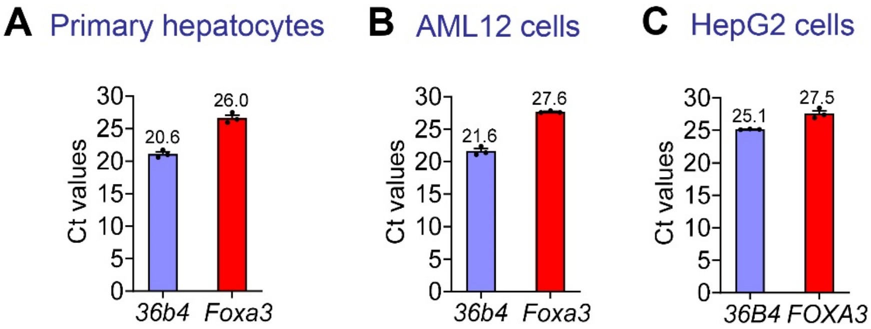

Supplementary Figure 1. FOXA3 expression in hepatocytes

A: *Foxa3* and *36b4* mRNA levels were determined in primary hepatocytes and expressed at *Ct* values (n=3). B: *Foxa3* and *36b4* mRNA levels were determined in AML12 cells and expressed at *Ct* values (n=3). C: *FOXA3* and *36B4* mRNA levels were determined in HepG2 cells and expressed at *Ct* values (n=3).

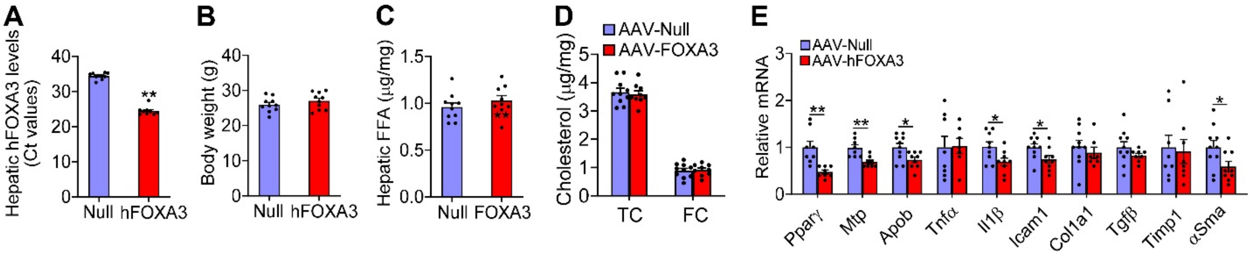

Supplementary Figure 2. Overexpression of hepatic FOXA3 does not affect body weight, hepatic FFA levels, or cholesterol levels but regulates hepatic gene expression in chow-fed mice

C57BL/6 mice were i.v. injected with AAV8-ALB-Null or AAV8-ALB-FOXA3 (n=10). After 4 weeks, mice were euthanized. A: Hepatic human *FOXA3* mRNA levels (*Ct* values). B: Body weight. C: Hepatic FFA levels. D: Hepatic TC and FC levels. E: Hepatic mRNA levels. A Student *t*-test was used for statistical analysis. **P*<0.05, ***P*<0.01

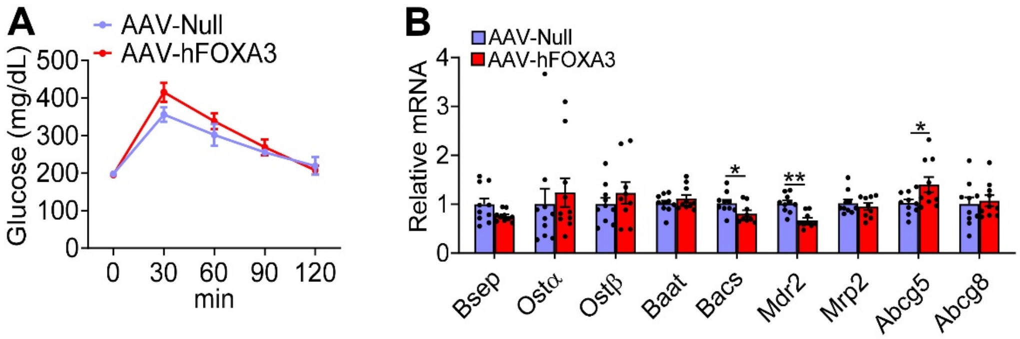

Supplementary Figure 3. Effects of hepatic FOXA3 overexpression on glucose tolerance or hepatic gene expression

C57BL/6 mice were i.v. injected with AAV8-ALB-Null or AAV8-ALB-FOXA3 and fed a Western diet for 16 weeks. A**:** Glucose tolerance test was performed (n=7-8) and no statistical significance between the two groups was observed. B: Hepatic mRNA levels (n=10). A two-way ANOVA (A) or student *t*-test was used for statistical analysis (B). *P<0.05, **P<0.01

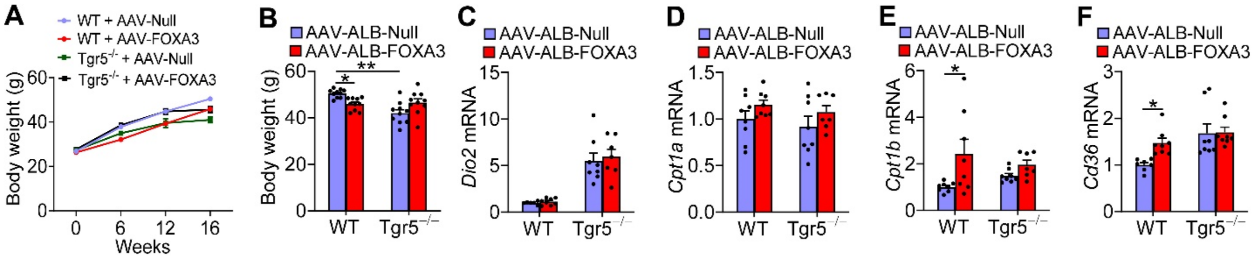

Supplementary Figure 4. Body weight and gene expression in brown adipose tissue of wild-type or *Tgr5*$^{-/-}$ mice overexpressing hepatic FOXA3

Wild-type (WT) and *Tgr5*$^{-/-}$ mice were i.v. injected with AAV8-ALB-Null or AAV8-ALB-FOXA3 and then fed a Western diet for 16 weeks (n=8-10). A-B: Body weight change over 16 weeks (A) and body weight at 16 weeks (B). C-F: mRNA levels of *Dio2* (C), *Cpt1a* (D), *Cpt1b* (E), and *Cd36* (F) in brown adipose tissue were determined. A two-way ANOVA was used for statistical analysis. *P<0.05, **P<0.01
